# Organ-on-chip technology: Opportunities and challenges

**DOI:** 10.1016/j.biotno.2024.01.001

**Published:** 2024-01-05

**Authors:** Santosh Kumar Srivastava, Guo Wei Foo, Nikhil Aggarwal, Matthew Wook Chang

**Affiliations:** a NUS Synthetic Biology for Clinical and Technological Innovation (SynCTI), National University of Singapore, Singapore; b Synthetic Biology Translational Research Programme, Yong Loo Lin School of Medicine, National University of Singapore, Singapore; c Department of Biochemistry, Yong Loo Lin School of Medicine, National University of Singapore, Singapore; d National Centre for Engineering Biology (NCEB), Singapore

**Keywords:** 2D/3D culture models, Organs-on-chips (OOCs), Microfluidics, Lung-on-chip, Kidney-on-chip, Liver-on-chip, Gut-on-Chip

## Abstract

Organ-on-chip (OOC) technology is an innovative approach that reproduces human organ structures and functions on microfluidic platforms, offering detailed insights into intricate physiological processes. This technology provides unique advantages over conventional in vitro and in vivo models and thus has the potential to become the new standard for biomedical research and drug screening. In this mini-review, we compare OOCs with conventional models, highlighting their differences, and present several applications of OOCs in biomedical research. Additionally, we highlight advancements in OOC technology, particularly in developing multiorgan systems, and discuss the challenges and future directions of this field.

## 1 Introduction

The past five to six decades have witnessed significant scientific and technological advancements in biotechnology and biomedical research.^1^, ^2^, ^3^ This progress has deepened our understanding of disease mechanisms, led to the identification of novel drug targets, and facilitated biomarker discovery. Primary assessment of drug efficacy, toxicity, and cellular mechanisms in biomedical research is essential before clinical trials.^4^ However, traditional in vitro cell culture models lack physiological complexity, while in vivo animal models face ethical concerns, interspecies differences, and limited translatability.^5^ Recent breakthroughs in biomedical research, microfabrication, and microfluidics have paved the way for the development of micro-engineered models of functional units of human organs, known as organs-on-chips (OOCs). This innovative technology spans a spectrum of organ types, including the liver, lung, gut, kidney, brain, heart, and skin.^6^^,^^7^ By reproducing essential physiological attributes of human organs, OOCs possess the capacity to transform the landscape of drug discovery and personalized medicine. In this mini-review, we compare OOCs to conventional models, discuss examples of how OOCs have been used in drug development and understanding disease mechanisms, and explore the challenges and future directions of research on OOC.

## 2 Comparison of conventional models with organ–on–chip

**Fig. 1:**
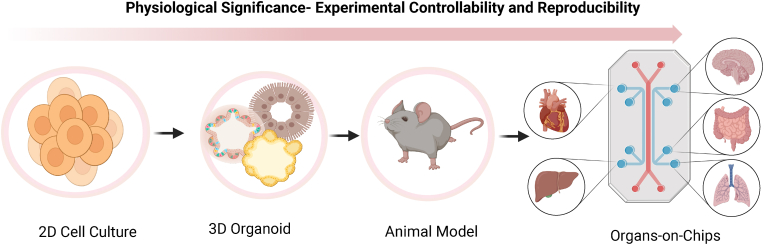
Illustration depicting preclinical models employed in biomedical research. Conventional 2D/3D cell cultures facilitate rapid and reliable drug response but their ability to fully replicate the physiology and pathology of living organs is limited. Animal models faithfully mimic physiological aspects but lack human compatibility. Microfluidic OOC platforms offer an experimentally controllable cell culture within an organotypic microarchitectural environment, providing a more physiologically significant platform.

Conventional models, such as animal models and cell culture systems, have played a vital role in biomedical research and drug development.^8^ In the realm of drug development, studying diseases, and safety assessment, OOCs offer compelling advantages over traditional 2D/3D cell culture models. In 2D cell cultures, cells are grown on a flat surface, while in 3D cell cultures, cells are grown in a three-dimensional space, typically embedded in a gel-like matrix or a sturdy scaffold.^9^ Although 2D culture models provide ample data at relatively low costs, they fall short in replicating the complex pathophysiology observed in human patients. This limitation necessitates computational modelling and systems biology approaches to predict in vivo drug responses.^10^ Biomimetic 3D tissue structures equipped with physiological barriers accurately simulate drug delivery and penetration in vivo compared to 2D cell monolayers in conventional culture models.^11^ However, 3D culture models are limited in their ability to replicate complex physiological functions at the organ level and clinically relevant disease presentations involving various tissue types, challenges that can be overcome by OOCs (Fig. 1). Additionally, OOCs offer the capability to regulate fluid movements, allowing the replication of a diverse range of mechanical stimuli, such as those induced by physiological flow (e.g., blood circulation and interstitial flow).^7^ Such fluid flow is not feasible in 3D culture models. This distinctive attribute of OOCs enables more comprehensive and precise predictions of intricate drug responses within living organisms.

OOCs also present notable advantages over existing animal models. Although animal studies can mimic physiological complexity at the whole-organism level, their validity and translatability to humans are increasingly questioned.^12^ Recent systematic evaluations of their predictive value have shown limited correlation between data obtained from animal studies and human outcomes. This is due to substantial interspecies differences in key disease pathways and gene expression profiles*5,.*^13^ This underscores the pressing need for innovative approaches to model complex human-relevant conditions. OOC systems confer a fundamental advantage over animal models, providing real-time visualization and quantitative, high-resolution analysis of a spectrum of human biological processes. Based on these unique advantages, OOCs have the potential to surpass conventional in vitro and in vivo models in enabling robust biomedical research and drug development.

## 3 Application of organ–on–chip in biomedical research

OOC technology represents groundbreaking innovation with profound implications for biomedical research and its applications in various organ systems.^14^ For example, in one study, the authors demonstrated the development of a biomimetic microsystem that accurately recreates the crucial alveolar-capillary interface of the human lung, enabling the visualization and characterization of inflammatory processes and responses to bacterial stimuli.^15^ In a recent study, a lung-on-chip model served as a vital tool for investigating the effects of SARS-CoV-2 infection. This study demonstrated that the virus can infect and damage the alveolar epithelium, the lining of the air sacs in the lungs, contributing to the respiratory complications associated with COVID-19.^16^^,^^17^

Microfluidic devices have also been used to cultivate hepatic cell lines in a three-dimensional format, resulting in the creation of a liver-on-a-chip model. In one study, the authors developed a multichannel liver-on-chip model to mimic the pathophysiology of non-alcoholic steatohepatitis (NASH).^18^ For this, the authors cultured hepatocytes, Kupffer cells, endothelial cells, and stellate cells in a microfluidic device and used a fatty-acid-rich medium to induce lipotoxicity. The model showed typical disease phenotype and can potentially be used for anti-NASH drug screening.^18^

The kidney-on-chip model was utilized to investigate drug-induced nephrotoxicity on kidney tubules. In such a model developed using primary human proximal tubule cells, cisplatin was found to induce cell injury when administered in the interstitial compartment of the microfluidic device. This toxicity by cisplatin was completely abolished by the co-administration of cimetidine, an inhibitor of the cation transporter responsible for cisplatin uptake.^19^

The gut-on-chip model has also been employed to investigate the impact of different treatments on gastrointestinal disorders. In a gut-on-chip study, the VSL#3 probiotics formulation, consisting of *Lactobacillus acidophilus, Lactobacillus plantarum, Lactobacillus paracasei, Bifidobacterium breve, Bifidobacterium longum, and Bifidobacterium infantis,* demonstrated a decrease in intestinal inflammation and an improvement in gut barrier function.*^20^* Another study harnessed the power of gut-on-chip to evaluate the treatment of NL63 coronavirus infection in the intestinal epithelium.*^21^* This study showed that although nafamostat inhibited NL63 infection, remdesivir was ineffective and even toxic to the endothelium. Overall, OOC technology is transforming biomedical research, providing insights into disease mechanisms, accelerating drug screening and development of personalized medicine and improving safety assessments.

## 4 Advanced organ–on–chip technology: multi-organ systems

**Fig. 2:**
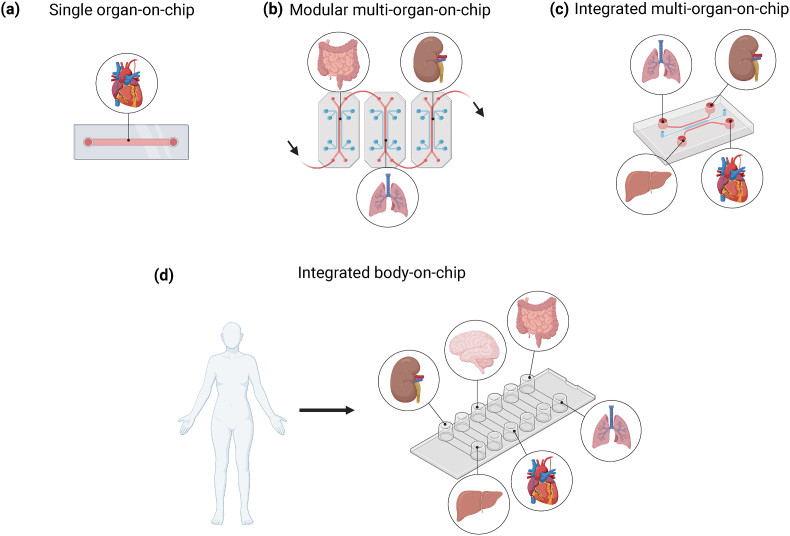
Illustration depicting various configurations of OOC technology: **(a)** A single OOC represents a standalone model, showcasing the isolation and study of an individual organ's microenvironment. **(b)** A schematic illustrating a modular MOC, demonstrating the interconnectedness of multiple OOC modules to study the interactions between different organs. **(c)** A diagram depicting an integrated MOC, exemplifying a unified platform where multiple organ systems are interconnected in a single chip, allowing for more comprehensive studies of physiological responses. **(d)** Visualization of an integrated body-on-chip, representing a holistic approach with multiple organs integrated into a chip to simulate the complexity of the human body. This provides a platform for advanced studies on drug response, personalized medicine, and disease mechanisms.

Although OOC can recapitulate the majority of single organ characteristics and physiological flow conditions (Fig. 2a), they are unable to capture the systemic interactions between multiple organs. These interactions require accurate modelling to understand the fate and distribution of drugs, identify biomarkers in bodily fluids, and determine the pathogenesis of multi-organ diseases.*^22^* While animal models are commonly used for elucidating such interactions, they suffer from several limitations discussed above. To fulfil this need for faithful models, efforts have been undertaken to integrate multiple OOCs into a single device, forming multi-organ-on-chip systems (MOC). MOC systems represent a significant stride towards creating more realistic and physiologically relevant in vitro models.

Currently, there are two types of MOC platforms: (i) a modular system where multiple single-OOC are connected, and (ii) different organs integrated into the same device connected via channels*^22^* (Fig. 2b and c). As an example of the former configuration, the authors developed a serially connected liver-on-chip, heart-on-chip, and lung-on-chip to analyse inter-organ response to drug administration.*^23^* The authors showed that upon administration of bleomycin, a known lung toxin, even the cardiac organoids showed adverse effects. This was attributed to the release of cardiotoxic inflammatory cytokines from the lungs due to bleomycin administration. For the second type of MOC platform, a heart-liver-skin integrated system was developed to study the effects of acute and chronic exposure to drug.*^24^* Using their system, the authors could compare the responses to drugs applied topically versus those administered systemically.

Beyond two- and three-organs-on-chip systems, attempts have been made to capture the complete body-on-chip (Fig. 2d). In one example, the authors developed a platform linking gut-, liver-, heart-, kidney-, lung-, skin-, brain-, and blood brain barrier-on-chips with vascular channels.*^25^* This platform was combined with a liquid-handling robotic and mobile microscope for automated culturing, medium addition, sample collection, and imaging. This interconnected system represents a comprehensive emulation of the human body, reflecting the intricate network of physiological processes and inter-organ communication.

## 5 Challenges and future directions

There are several challenges associated with OOC and MOC technology, despite its innovative nature. One prominent challenge is the development of a blood mimetic, a universal medium with nutrients and growth factors for the maintenance of different cell types. In some cases, a 1:1 ratio of different media has been used; however, with the advent of complex MOCs, this problem is further compounded.*^26^* Another critical challenge in OOC technology and MOC is the standardization of the manufacturing process.*^27^* Currently, there is a lack of universally accepted protocols and materials for creating OOC devices. The absence of standardization not only hinders the reproducibility of experiments but also acts as a bottleneck for large-scale, cost-effective production. Establishing standardized materials and fabrication techniques is essential for fostering collaboration and accelerating the adoption of OOC technology across different research laboratories. Furthermore, the intricate balance of organ sizes, inter-organ transportation rates, and the liquid-to-cell ratio presents a multifaceted challenge. Achieving physiologically relevant conditions within the chip requires a meticulous understanding of these parameters. The challenge lies not only in replicating the physical characteristics of individual organs but also in emulating their dynamic interactions, transport phenomena, and response to stimuli. The need for experimental conditions that mirror real physiological processes while remaining technically feasible poses a continuous challenge in the development and application of OOC technology.

**Fig. 3:**
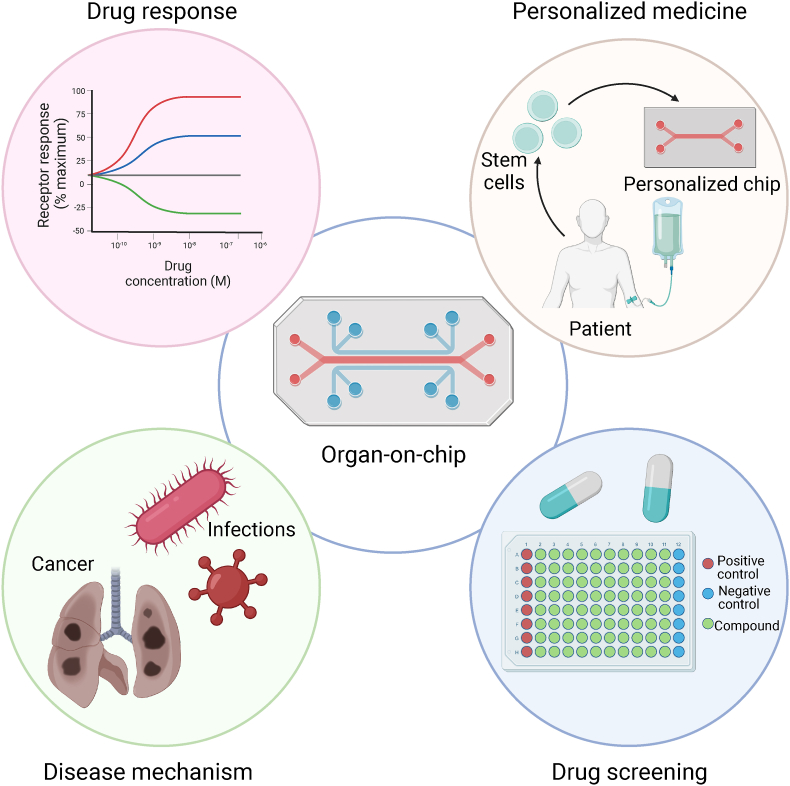
Schematic diagram depicting the multifaceted opportunities offered by OOC technology in the realms of drug response, personalized medicine, elucidation of disease mechanisms, and drug screening. The figure showcases the potential versatility of OOC platform in mimicking human organ microenvironments, enabling drug screening, studying drug responses and doses tailored to individual patients, and providing insights into the intricate mechanisms underlying diseases. This integrated approach holds the potential for advancing precision medicine and accelerating the drug discovery process.

Despite these challenges, OOC technology continues to develop at a rapid pace, and the future holds much promise for its continued advancement. The evolving landscape of OOC and MOC technology aligns synergistically with the changing paradigms of regulatory bodies, exemplified by the US Food and Drug Administration's progressive shift away from mandatory animal testing towards more advanced and ethical methodologies.^28^ The European Organ-on-Chip Society (EUROoCS) and the U.K. Organ-on-a-Chip Technologies Network play pivotal roles in connecting academic innovators with regulatory and industry input.^29^^,^^30^ Many other national OOC networks are also emerging, reflecting the global momentum in this field. OOCs stand poised to become the standard for biomarker discovery and drug screening, leveraging their potential to personalize chips using patient-derived cells, particularly in the realm of rare genetic disorders. Integration of OOCs with sophisticated analytical instruments, liquid-handling systems, and advanced imaging technologies heralds the creation of intricate multi-organ models, offering a holistic emulation of complex systemic and inter-organ interactions. This convergence is poised to deepen our understanding of disease processes, pave the way for personalized medicine, and enhance the precision of drug efficacy assessments (Fig. 3).

In conclusion, OOC technology represents a promising approach toward more accurate and efficient biomedical research methodologies. The ongoing evolution of OOC technology aligns with the changing paradigms in biomedical research, offering a more accurate and efficient alternative to traditional in vitro and in vivo studies. Overcoming the challenges requires collaborative efforts to standardize protocols, optimize experimental conditions, and address technical intricacies. As these challenges are addressed, OOC and MOC technology stand poised to reshape the landscape of biomedical research, drug discovery, and personalized medicine, unlocking new possibilities for advancing human health.

## Declaration of competing interest

The authors declare no conflict of interest. Matthew Wook Chang is the Editor-in-Chief for Biotechnology Notes and was not involved in the editorial review or the decision to publish this article.
